# Evaluation of Autoimmune FVIII Inhibitor Using Clot Waveform Analysis in Emicizumab-Treated Patients

**DOI:** 10.3390/jcm15010271

**Published:** 2025-12-29

**Authors:** Shigehisa Tamaki, Hideo Wada, Naoki Shinke, Junichiro Nishiki, Ryota Sasao, Atsushi Fujieda, Takeshi Matsumoto, Isao Tawara, Teruto Hashiguchi

**Affiliations:** 1 Department of Hematology, Japanese Red Cross Ise Hospital, 471-2 Hunae-Ichome, Ise 516-8512, Mie, Japan; stamaki@ise.jrc.or.jp (S.T.); n-shinke@clin.medic.mie-u.ac.jp (N.S.); cerpurf@gmail.com (J.N.); 312041ryouta@gmail.com (R.S.); fujieda2001@nifty.com (A.F.); 2 Department of General Medicine, Mie Prefectural General Medical Center, 5450-132 Ohaza Hinaga, Yokkaichi 510-8561, Mie, Japan; 3 Department of Transfusion Medicine and Cell Therapy, Saitama Medical Center, Saitama Medical University, 1981 Kamoda, Kawagoe 350-8550, Saitama, Japan; matsutak@saitama-med.ac.jp; 4 Department of Hematology and Oncology, Mie University Graduate School of Medicine, Tsu 514-8507, Mie, Japan; itawara@clin.medic.mie-u.ac.jp; 5 Department of Laboratory and Vascular Medicine, Cardiovascular and Respiratory Disorders, Kagoshima University Graduate School of Medical and Dental Sciences, 8-35-1 Sakuragaoka, Kagoshima 890-8520, Japan; k1581347@kadai.jp

**Keywords:** autoimmune factor VIII deficiency, FVIII, inhibitor, clot waveform analysis, emicizumab

## Abstract

**Background/Objectives**: Autoimmune factor VIII deficiency (AiFVIIID) is a rare disorder that causes severe bleeding. Emicizumab has recently been found to be effective in treating AiFVIIID; however, monitoring with standard coagulation tests presents challenges. **Methods**: Clot waveform analysis (CWA), which involves CWA-activated partial thromboplastin time (APTT), the CWA-small amount of tissue factor activation assay (sTF/FIXa), and clotting time using a small amount of thrombin (sTT), was used to both diagnose AiFVIIID and monitor emicizumab. **Results**: CWA-sTT reflects the residual FVIII activity in patients with AiFVIIID. Several tests were employed, including APTT, FVIII activity, CWA, mixing tests with normal plasma, FVIII inhibitor assays, and anti-FVIII antibody activity for the diagnosis of AiFVIID in three cases. However, the sensitivity of APTT reagents to AiFVIID differed between thrombocheck-APTT and APTT-SP. Emicizumab treatment was effective for major bleeding, and anti-FVIII antibody activity could be measured using CWA-sTT. **Conclusions:** The sensitivity of APTT reagents to AiFVIID varies. CWA-sTT may provide utility in the diagnosis of AiFVIIID. Emicizumab is useful for the treatment of AiFVIID, and anti-FVIII antibody activity can be measured even in patients treated with emicizumab.

## 1. Introduction

Autoimmune factor VIII (FVIII) deficiency (AiFVIIID), commonly known as acquired hemophilia A [1,2,3,4], is a rare disorder caused by autoantibodies against endogenous FVIII, which, in some cases, can lead to severe bleeding. AiFVIIID is more common in elderly patients, with about half of AiFVIIID cases being idiopathic and the remainder being associated with autoimmune diseases, malignancies, or infections [4,5]. In recent years, numerous cases of AiFVIIID related to COVID-19 vaccination [6] or infection [7] have been reported. Tests to differentiate AiFVIIID from hemophilia A without inhibitors [8,9] or lupus anticoagulant [10,11] are vital for diagnosing AiFVIIID [3,12].

The treatment of AiFVIIID includes the use of immunosuppressive therapy to suppress autoantibody production [3,13,14,15] and bypass therapy to control bleeding [16,17,18,19,20]. Immunosuppressive therapy [3,13,14,15] includes the use of corticosteroids, rituximab, and cyclophosphamide, while bypass therapy includes the use of recombinant activated factor VII (rFVIIa) [16], activated prothrombin complex concentrate (APCC) [17], emicizumab (Chugai Pharmaceutical, Tokyo) [18,19], and tissue factor pathway inhibitor (TFPI) antibodies [20]. It is difficult to evaluate the effectiveness of bypass therapy using routine coagulation tests such as activated partial thromboplastin time (APTT) or prothrombin time (PT). Therefore, thromboelastography (TEG) or thrombin generation tests (TGTs) are often used to monitor bypass therapy in AiFVIIID [21,22].

Emicizumab mimics the activity of FVIII and is a humanized bispecific monoclonal antibody targeting FIX or FIXa and FX [23]. It has been reported that patients treated with emicizumab exhibit significantly shortened APTT [24]. Therefore, APTT cannot be used to monitor patients treated with emicizumab, nor can it be used to measure inhibitory titers. It has been reported that FVIII activity in these patients can be measured using neutralizing antibodies against emicizumab [25,26]. Additionally, FVIII activity can be measured using a clot waveform analysis (CWA), with clotting time determined using a small amount of thrombin (CWA-sTT) [27].

In this study, we investigated hemostatic abnormalities in three patients with AiFVIIID and measured anti-FVIII antibody activity in patients treated with emicizumab using CWA-sTT.

## 2. Materials and Methods

Between 4 January 2023, and 30 April 2025, three patients with AiFVIIID were admitted to the Hematology Department of Ise Red Cross Hospital, Japanese Red Cross Society (Table 1). These patients exhibited severe bleeding tendencies and remarkable hematological abnormalities, and following AiFVIIID diagnosis, they received bypass and immunosuppressive therapies. The study protocol (H2022-221) was approved by the Mie University Ethics Committee, and written informed consent was obtained from each participant. This study was conducted in accordance with the principles of the Declaration of Helsinki.

The reagents and instruments used were as follows: thrombocheck APTT (Sysmex Corporation, Kobe, Japan); platelet-rich plasma (PRP, laboratory-prepared), which was prepared via centrifugation at 900 rpm for 15 min (platelet count, 40 × 10^10^/L); platelet-poor plasma (PPP, laboratory-prepared), which was prepared via centrifugation at 3000 rpm for 15 min (platelet count, <0.5 × 10^10^/L) [28]; normal pooled PPP (laboratory-prepared); HemosIL APTT-SP (Werfen Japan, Tokyo, Japan); FVIII-deficient plasma (Werfen); thrombin (Thrombin 500 units, Mochida Pharmaceutical Co., Ltd., Tokyo, Japan); HemosIL RecombiPlasTin 2G (Werfen); anti-FVIII antibody, which was kindly provided by Chugai Pharmaceutical Co., Ltd. (Tokyo, Japan); and an ACL-TOP^®^ system (Werfen).

APTT was measured using patient PPP, thrombocheck APTT and a fully automated blood coagulation time measurement device. Cross-mixing tests of APTT with normal pooled PPP from 20 healthy volunteers for inhibitor detection were performed using thrombocheck-APTT or APTT-SP. FVIII activity and FVIII inhibitors without emicizumab were measured using a one-step clotting assay with APTT at SRL, Inc. (Tokyo, Japan). FVIII inhibitor titer with emicizumab was kindly measured using anti-idiotype monoclonal antibodies against emicizumab [26] by Chugai Pharmaceutical Co., Ltd.

**Figure 1:**
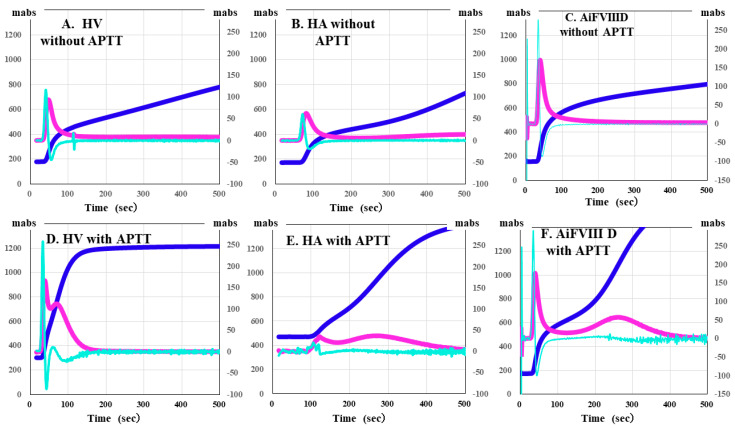
Clot waveform analysis of clotting time determined using a small amount of thrombin. (**A**,**D**) healthy volunteer (HV); (**B**,**E**) hemophilia A (HA); (**C**,**F**) autoimmune factor VIII deficiency (AiFVIIID); (**A**–**C**) without APTT reagent; (**D**–**F**) with APTT reagent; APTT, activated partial thromboplastin time; navy line, fibrin formation curve; pink line, first derivative curve (velocity); light blue, second derivative curve (acceleration). In HV, the thrombin burst is enhanced by the addition of APTT reagent, whereas in HA, it is not enhanced, and AiFVIIID is slightly enhanced.

CWA-APTT was measured using patient PPP and HemosIL APTT-SP on an ACL-TOP system based on previously reported methods [29]. The CWA-small amount of tissue factor activation assay (sTF/FIXa) was performed using patient PRP and 2000-fold diluted HemosIL RecombiPlasTin 2G with saline solution including CaCl_2_ on an ACL-TOP^®^ system [30]. The CWA-sTT of patient PPP was measured using an ACL-TOP^®^ system with 0.2 IU of thrombin, which was diluted with 0.9% saline solution including CaCl_2_ [26,29]. CWA-sTT was measured with or without 16-fold diluted APTT-SP with 0.9% saline solution. CWA-sTT reflects thrombin burst caused by activated platelets and activated FVIII (FVIIIa), and when APTT reagent is added, it is more strongly affected by FVIIIa. In hemophilia A (HA), since the residual FVIII is extremely low, CWA-sTT is only minimally enhanced; in AiFVIIID, in comparison, it shows mild enhancement (Figure 1).

In the CWA-sTT, a small amount of FVIIIa is generated, which counteracts the effect of emicizumab, and since there is no incubation with the APTT reagent, it is considered not to be affected by emicizumab. In the CWA, three types of curves are shown [26,27]. The first represents the change in absorbance observed during the TT measurement, corresponding to the fibrin formation curve (FFC). The second is the first derivative of the absorbance peak (first DP), corresponding to the coagulation velocity. The third is the second derivative of the absorbance peak (second DP), corresponding to the coagulation acceleration.

**Figure 2:**
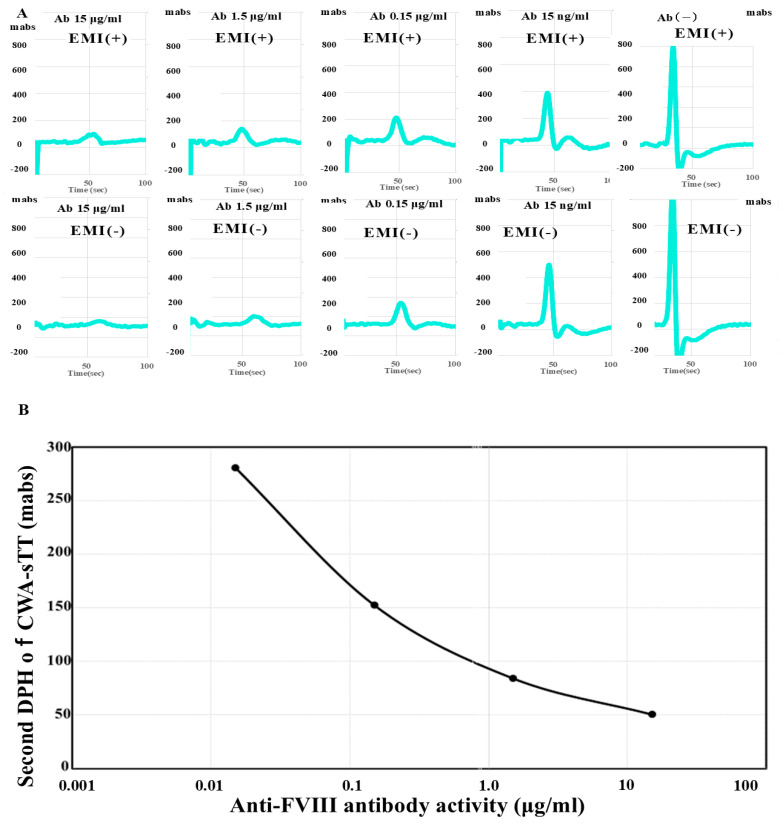
CWA-sTT for various anti-FVIII antibody concentrations (**A**) and Standard curve for anti-FVIII antibody concentration using CWA-sTT. (**A**): Upper section, with emicizumab; lower section, without emicizumab; CWA-sTT, clot waveform analysis–clotting time using a small amount of thrombin; FVIII, clotting factor VIII; Ab, antibody; EMI, emicizumab; DPH, derivative peak height; light blue, second derivative curve (acceleration); Regardless of the presence of emicizumab, CWA-sTT shows a pattern dependent on anti-FVIII antibody levels; CWA-sTT can be used to measure anti-FVIII antibody titers in the presence of emicizumab. (**B**): Standard curve for the anti-FVIII antibody activity. FVIII activity in the presence of emicizumab was measured using CWA-sTT as previously reported [26]. The second derivative peak height of CWA-sTT in normal plasma diluted tenfold with FVIII-deficient plasma shows linearity across FVIII levels from 0.1% to 100%.

Anti-FVIII antibody activity: Measuring FVIII activity using CWA-sTT enables sensitive detection of residual FVIII activity in patients with AiFVIIID. The measured residual FVIII activity was converted into the amount of FVIII antibody added to normal plasma. Because CWA-sTT shows a similar pattern in normal plasma regardless of the presence of emicizumab, it can be used to measure FVIII activity in the presence of emicizumab. When anti-FVIII antibodies were added, the peak height of the second derivative curve in the CWA-sTT during FVIII activity measurement correlated with the anti-FVIII antibodies in a dose-dependent manner. Therefore, in the FVIII activity measurement system using CWA-sTT, anti-FVIII antibody activity could be evaluated in the range of 0.02 μg/mL to 10 μg/mL regardless of the presence of emicizumab (Figure 2).

## 3. Results

**Figure 3:**
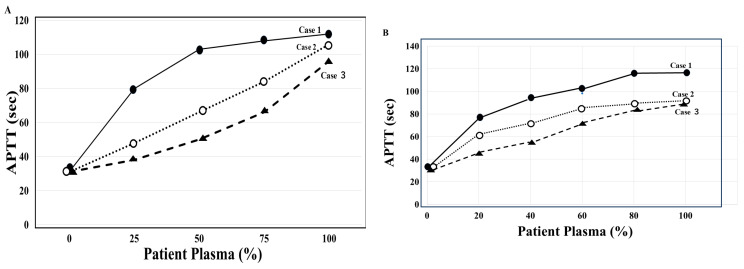
Cross-mixing test of APTT using normal plasma with thrombocheck APTT (**A**) and APTT-SP (**B**) in three cases of AiFVIIID. APTT using thrombocheck-APTT shows a convex upward trend in case 1 but a concave downward trend in case 3. APTT using APTT-SP shows a convex upward trend in cases 1–3, suggesting that the three patients tested positive for inhibitors against the coagulation factor. AiFVIIID, autoimmune coagulation factor VIII (FVIII) deficiency; APTT, activated partial thromboplastin time; closed circle, case 1; open circle, case 2; closed triangle, case 3.

Case 1: The first case comprises a 62-year-old male (Table 1), with the chief complaint of hematoma of the left thigh. His past and family histories showed no bleeding tendencies. The patient was hospitalized due to multiple fractures and nerve injuries from a traffic accident. Two months after the injury, he developed hydrocephalus and underwent VP (ventriculoperitoneal) shunt surgery. Subsequently, he contracted COVID-19 and received treatment, including remdesivir (Gilead Sciences, Foster City, CA, USA), at a rehabilitation hospital to which he had been transferred. Two weeks after the onset of COVID-19, he was admitted to the Hematology Department due to general fatigue and the Appearance of a hematoma on his left thigh, with hemoglobin levels dropping to 4.8 g/dL. Based on prolonged APTT (105 s), FVIII activity < 1%, an inhibitor pattern on a mixing test with normal plasma (both thrombocheck APTT and APTT-SP, Figure 3), and an FVIII inhibitor level of 37.3 Bethesda units (BU)/mL, he was diagnosed with AAFVIIID. He remained stable without bleeding symptoms on prednisolone (PSL, Shionogi & Co., Ltd., Osaka, Japan) monotherapy. Activity recovered to approximately 20–30%, and the patient completed treatment and returned to normal daily activities.

**Figure 4:**
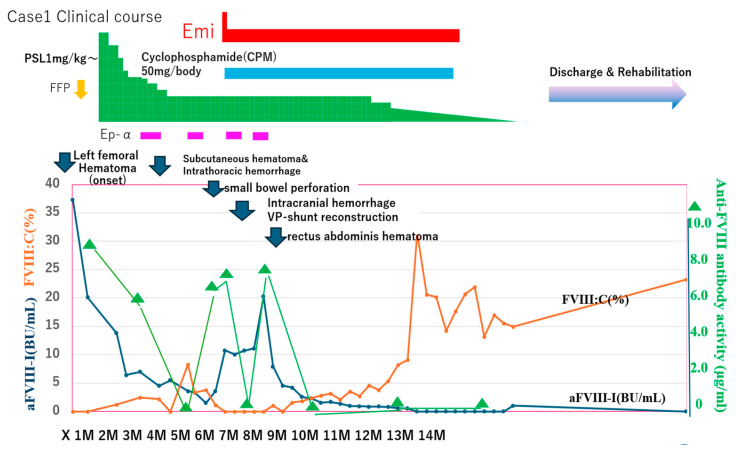
Clinical course, and FVIII activity and anti-FVIII antibody in case 1” to “FVIII activity, anti-FVIII antibody and anti-FVIII antibody activity in clinical course of case 1. Emi, emicizumab; Ep-α, eptacog alfa; FVIIa, activated clotting factor VII; PSL, prednisolone; FVIII, clotting factor VIII; FVIII:C, FVIII clotting activity; aFIII-I, anti-FVIII inhibitor; BU, Bethesda units; GI, gastrointestinal.

**Figure 5:**
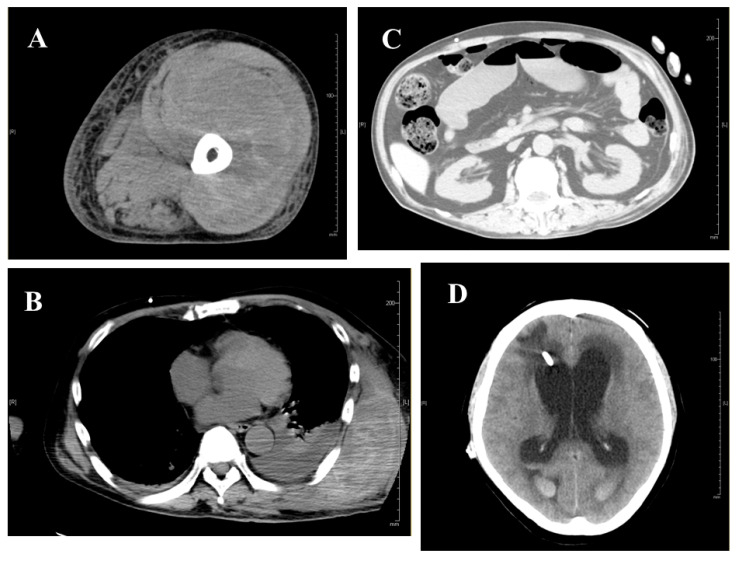
(**A**) CT image of a left femoral hematoma at disease onset. (**B**) CT findings of subcutaneous hematoma and intrathoracic hemorrhage on day 56 after admission. (**C**) CT image demonstrating strangulated ileus complicated by small bowel perforation, requiring laparotomy on day 80 after admission. (**D**) CT findings of intracranial hemorrhage on day 102 after admission.

Two and a half months after hospitalization in the Hematology Department, a large subcutaneous hematoma appeared on his left back and chest, complicated by small bowel obstruction (Figure 4 and Figure 5). Eptacog alpha (Ep-α; rhFVIIa, Novo Nordisk, Bagsværd, Denmark) was administered for small intestine resection and massive bleeding. Due to persistent APTT prolongation, emicizumab was administered 3.2 months after hospitalization to achieve stability.

Case 2: The second case comprises a 71-year-old male (Table 1) with a chief complaint of walking difficulty due to right lower limb pain. His medical history showed head trauma and skull fracture (at age 19), traumatic epilepsy (from age 40), left chronic subdural hematoma (at age 69), lumbar spinal canal stenosis, hepatitis B virus infection, and no bleeding tendencies, and his family history showed no history of bleeding. The patient visited the emergency department because of walking difficulties due to bleeding and pain in all four limbs, and he was referred to the Hematology Department due to a prolonged APTT (85 s). FVIII activity was <1%, and a mixing test using APTT-SP with normal plasma showed an inhibition pattern. As FVIII inhibitors impact hemostatic control. hemostatic activity was monitored using CWA-sTT, which is not affected by emicizumab. When gastrointestinal bleeding, cerebral hemorrhage, and intraperitoneal bleeding occurred, the peak height of CWA-sTT significantly decreased. In addition to PSL, oral cyclophosphamide (CPM, Shionogi & Co., Ltd., Osaka, Japan) 50 mg/day was administered. Thereafter, 8.7 months after hospitalization, the anti-FVIII inhibitors became undetectable. As FVIII autoantibodies were detected, the patient was diagnosed with AiFVIIID (Figure 3). At the time of admission, he was treated with PSL and Ep-α, followed by emicizumab one week after admission. His bleeding symptoms improved, and he was discharged 23 days after admission. After discharge, the patient continued to receive emicizumab together with PSL prescriptions on an outpatient basis, and six weeks after hospitalization, CPM was added at a dose of 50 mg per day. CPM was continued for approximately 12 months, and PSL for about 15 months. FVIII activity remained below 1%, and high-titer FVIII inhibitors persisted; immunosuppressive therapy was discontinued, however. The patient is currently on self-administered emicizumab monotherapy and has returned to normal daily activities.

Case 3: The third case comprised a 73-year-old male (Table 1) with the chief complaint of a hematoma of the right thigh. His past medical and family histories showed cerebral infarction with no history of bleeding tendency. His comorbidities included essential thrombocythemia (ET) and chronic kidney disease. The patient was diagnosed with ET seven years before the onset of AiFVIIID and had been treated with hydroxyurea (Cheplapharm, Mesekenhage, Germany), anagrelide (Takeda Pharmaceutical Company, Tokyo, Japan), and low-dose aspirin (Bayer, Tokyo, Japan). The patient developed difficulty in walking due to a hematoma in the right thigh and was referred to in the Hematology Department.

Laboratory findings showed prolonged APTT, decreased FVIII activity, an inhibitor-type pattern in a mixing test using normal plasma with APTT-SP (Figure 3), and the presence of FVIII inhibitors, leading to a diagnosis of AAFVIIID. Combination therapy with emicizumab and prednisolone (PSL) was initiated, resulting in rapid improvements in both the thigh hematoma and coagulation abnormalities. The patient was discharged early and continued outpatient follow-up. FVIII inhibitors became undetectable after 2 months, FVIII activity recovered within 4 months, immunosuppressive therapy was completed within 5 months, and the patient was able to resume normal social activities.

CWA Evaluation

(1) CWA

**Figure 6:**
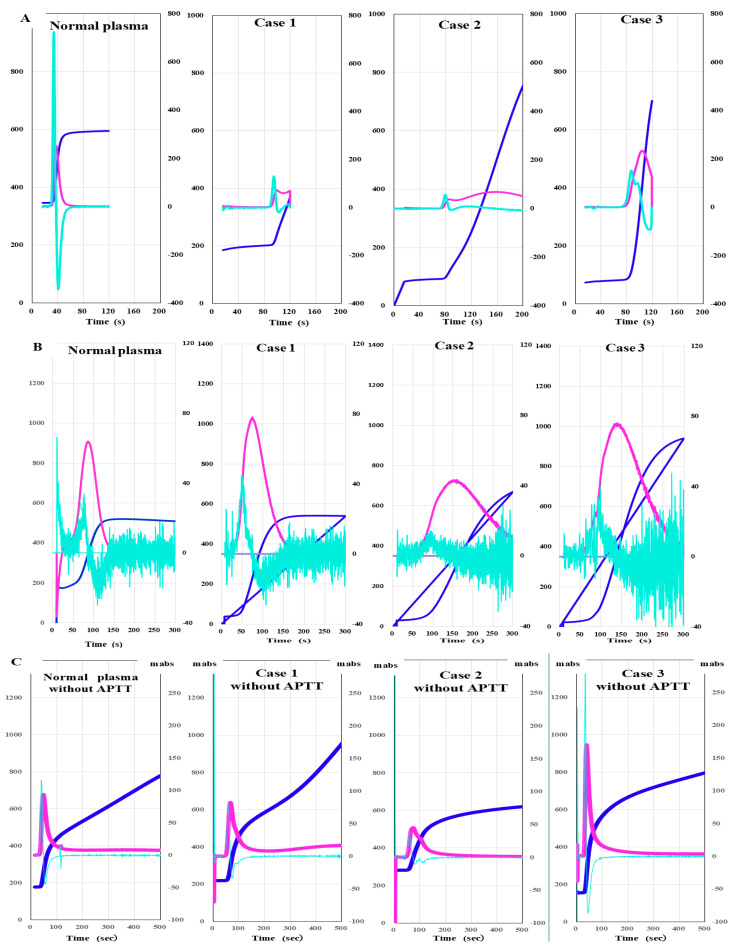

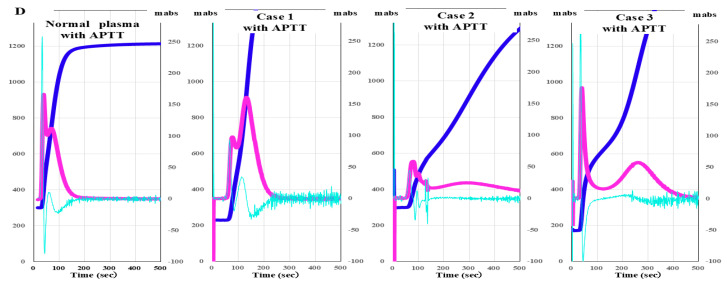
Clot waveform analysis of APTT (**A**), sTF/FIXa (**B**) and sTT without APTT reagent (**C**) and sTT with APTT reagent (**D**) in three cases of autoimmune factor VIII deficiency. APTT, activated partial thromboplastin time; sTF/FIXa, small amount of tissue factor- induced coagulation factor IX activation assay; sTT, clotting time using a small amount of thrombin; navy line, fibrin formation curve; pink line, first derivative curve (velocity); light blue, second derivative curve (acceleration). CWA-APTT was markedly abnormal in cases 1–3, CWA-STF/FIXa showed no significant difference among the 3 cases. CWA-sTT showed prolonged peak time and decreased peak height in cases 1–3 compared with normal plasma.

The peak time of CWA-APTT was significantly prolonged, and the peak heights of the first and second derivatives of CWA-APTT were markedly lower in cases 1, 2, and 3 than in normal plasma (Figure 6A). There was no significant difference in the peak time or height of CWA-sTF/FIXa between normal plasma and case 1. The peak time of CWA-sTF/FIXa was prolonged in cases 2 and 3 (Figure 6B). In CWA-sTT without APTT reagent, the peak time was prolonged and the peak height was decreased in cases 1 and 2. In CWA- sTT with APTT reagent, the peak time was prolonged, and the peak height was decreased in cases 1–3 (Figure 6C,D).

(2) Anti-FVIII antibody activity in AAFVIII patients (case 1) treated with emicizumab. CWA-sTT could be used to measure anti-FVIII antibody activity in patients treated with emicizumab. Of note, anti-FVIII antibody activity increased significantly during major bleeding and decreased after FVIIa treatment (Figure 4).

## 4. Discussion

AiFVIIID is diagnosed based on the absence of family and medical histories of bleeding tendency, the potential for massive bleeding, marked prolongation of APTT, significantly reduced FVIII activity, an inhibitor pattern on a mixing test with normal plasma, and confirmation of FVIII inhibitor titers [31,32]. Marked prolongation of APTT and a significant reduction in FVIII activity are initial effective diagnostic measures for suspected AiFVIIID; however, differentiation from hemophilia A is challenging yet essential [3,12]. Therefore, a definitive diagnosis of AiFVIIID is made using FVIII inhibitor titer measurement and a mixing test with normal plasma. In this study (Table 1), inhibitor titers were high in cases 1 and 2; in comparison, those in case 3 were not markedly high. The inhibitor pattern in a mixing test with normal plasma using APTT generally shows an upward convex curve [33]. In this study, an inhibitor pattern was observed in cases 1–3 in a mixing test using APTT-SP; however, when thrombocheck-APTT was used, case 3 did not show an inhibitor pattern. These findings suggest that APTT-SP may be more sensitive than thrombocheck-APTT in diagnosing inhibitors using a mixing test. There are also reports on the effects of APTT reagents on the sensitivity of LA or anticoagulants, making the choice of APTT reagent important for diagnosing AiFVIIID [34,35]. Furthermore, case 3 was complicated by accompanying ET, which could have potentially caused secondary von Willebrand disease [36], and as a result, the mixing test may have shown a factor deficiency pattern.

**Figure 7:**
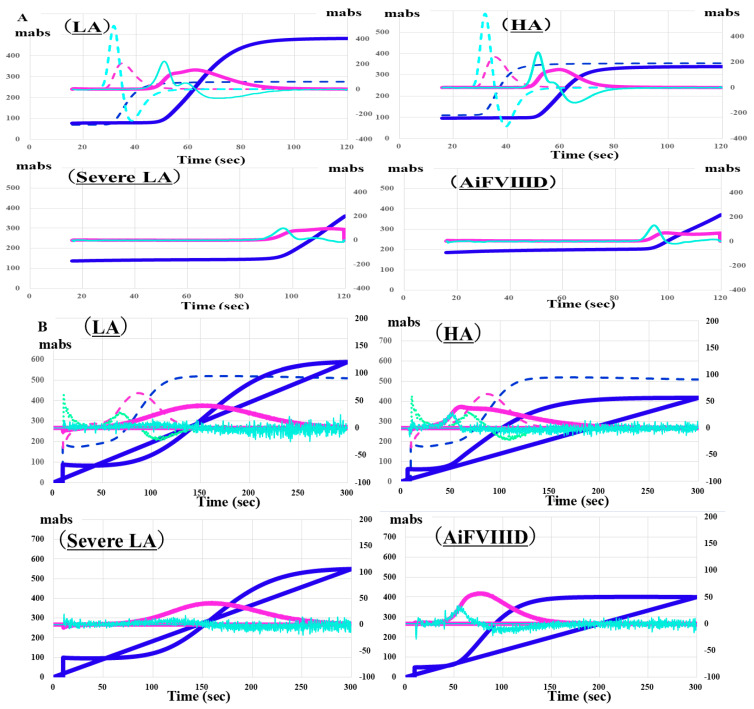
Clot waveform analysis of APTT (**A**) and sTF/FIXa (**B**) in patients with LA, severe LA, HA and AiFVIIID. Although the peak times of CWA-APTT are prolonged in patients with LA, severe LA, HA, and AiFVIIID, they are not significantly prolonged in patients with HA and AiFVIIID. APTT, activated partial thromboplastin time; sTF/FIXa, small amount of tissue factor-induced coagulation factor IX activation assay; LA, lupus anticoagulant; HA, hemophilia A; AiFVIIID, acquired autoimmune FVIII deficiency; navy line, fibrin formation curve; pink line, first derivative curve (velocity); light blue, second derivative curve (acceleration); solid line, patient; dotted line, control. CWA-APPT showed a similar pattern between LA and HA and between severe LA and AiFVIIID, and CWA-sTF/FIXa showed a similar pattern between LA and severe LA and between HA and AiFVIIID.

Regarding the CWA, no significant differences were observed in CWA-APTT between patients with hemophilia A and those with moderate LA or between AiFVIIID and those with severe LA [27]. PT was prolonged in patients with LA or severe LA, but not in patients with AiFVIIID or hemophilia A, suggesting that PT or sTF/FIX might be useful for distinguishing between LA and AiFVIIID [37] (Figure 7). A markedly low peak height of CWA-APTT was also effective in differentiating AiFVIIID from hemophilia A and LA [38]. It has been reported that a decrease in the peak height of CWA-APTT is more effective than an extension of the peak time in CWA-APTT for diagnosing hemophilia A or LA and predicting bleeding risk [39,40].

Bypass therapy for patients with inhibitors includes the use of APCC [41], rhFVIIa [42], emicizumab [43], and anti-TFPI antibodies [44]. Even in the presence of inhibitors against FVIII, APCC can promote hemostasis by acting downstream of FVIII through excess FVIIa, FIXa, FXa, or FIIa; in comparison, rhFVIIa promotes hemostasis via an extrinsic pathway and thrombin burst mechanism mediated by activated platelets through a large amount of FVIIa [45]. It has been reported that APCC or rhFVIIa should be monitored using CWA-APTT or CWA-sTF/FIXa, respectively [46]. Emicizumab is a bispecific antibody that binds simultaneously to FIXa and FX, exerting FVIIIa-like activity to achieve hemostasis [47,48].

In patients with inhibitors, bypass therapy including emicizumab can be effectively monitored using methods such as TEG [49] and TGT [50]. These methods are not feasible in general laboratories, however, and are expensive; they are not commonly used by general clinicians. CWA, which is a cost-effective and simple method, is useful for monitoring APCC and rhFVIIa. Emicizumab impacts the accuracy of APTT and FVIII assays; however, FVIII activity can be measured in the presence of emicizumab using neutralizing antibodies [51,52]. Furthermore, it has become possible to measure anti-FVIII antibody levels using an enzyme-linked immunosorbent assay (ELISA) [53,54]. However, measuring FVIII antibody levels with neutralizing antibodies or ELISA presents challenges. Therefore, measuring FVIII antibody activity using CWA-sTT may be useful for clinicians because it is straightforward and inexpensive.

Emicizumab is used in patients with hemophilia A and FVIII inhibitors and is widely administered because of its ease of dosing. However, emicizumab shortens the APTT beyond the level of FVIII activity, making it impossible to monitor true hemostatic function (FVIII activity) in patients using emicizumab. In other words, during the incubation period with APTT reagents in the APTT measurement system, the reaction between FIXa and FX progresses in the presence of emicizumab, significantly shortening the APTT. In contrast, CWA-sTT can be used to measure FVIII activity without being affected by emicizumab, enabling the assessment of coagulation function in patients receiving emicizumab.

## 5. Conclusions

In this study, we examined three cases of AiFVIIID that were controlled with immunosuppressive therapy such as steroids, in addition to bypass therapy including emicizumab or FVIIa. Mixing tests with normal plasma are useful for diagnosing AiFVIIID; however, as the sensitivity of APTT reagents to inhibitors varies, the results differ depending on the APTT reagent used. CWA-sTT reflects residual FVIII; thus, it may be useful in differentially diagnosing AiFVIIID from hemophilia A. Coagulation abnormalities in the presence of emicizumab cannot be assessed using APTT; however, they could be monitored using CWA-sTT. Anti-FVIII inhibitor titers are useful for diagnosing AiFVIIID, but they cannot be measured using the APTT method in the presence of emicizumab. By using CWA-sTT, it may be possible to readily measure FVIII antibody activity in the presence of emicizumab.

## Figures and Tables

**Table 1:** Patients with autoimmune coagulation factor FVIII deficiency.

	Case 1	Case 2	Case 3
Age	62	71	73
Sex	Male	Male	Male
Underlying disease	COVID-19	Posttraumatic epilepsy	MPN
Pre-existing condition	Posttraumatic hydrocephalus Ventriculoperitoneal shunt	Chronic subdural hematoma Resolved HBV infection	Transient ischemic attack Resolved HBV infection
Bleeding site (Onset)	Hematoma in left femoral region	Right lower limb Right pelvic region	Hematoma in right femoral region
Bleeding site (Clinical course)	Small bowel perforation Intraventricular hemorrhage Rectus sheath hematoma	None	None
APTT (s)	105	85	64
PT-INR	1.14	1.07	1.24
FVIII (%)	<1	<1	1.4
FVIII inhibitor (BU/mL)	37.3	174.1	2.1
Anti-FVIII ab activity (μg/mL)	8.7	6.6	1.2

APTT, activated partial thromboplastin time; PT-INR, prothrombin time-international normalized ratio; FVIII, clotting factor FVIII; BU, Bethesda units; anti-FVIII ab, anti-FVIII antibody; MPN, Myeloproliferative neoplasm; HBV, hepatitis B virus.

## Data Availability

The data presented in this study are available from the corresponding author upon request. The data are not publicly available due to privacy restrictions.

## Abbreviations

The following abbreviations are used in this manuscript:

AiFVIIID	autoimmune factor VIII deficiency
CWA	clot waveform analysis
APTT	activated partial thromboplastin time
sTT	clotting time using a small amount of thrombin
rFVIIa	recombinant factor VII
TFPI	tissue factor pathway inhibitor
APCC	activated prothrombin complex concentrate
PT	prothrombin time
TEG	thromboelastography
TGT	thrombin generation tests
FFC	fibrin formation curve
1st DP	first derivative of absorbance peak
2nd DP	second derivative of absorbance peak
PPP	platelet-poor plasma
PRP	platelet-rich plasma
sTF/FIXa	small amount of tissue factor activation assay
Ep-α	eptacog alpha
PSL	prednisolone
BU	Bethesda units
CPM	cyclophosphamide
ET	essential thrombocythemia
